# *N*,*N*′-Dimethyl-*N*,*N*′-diphenyl-3-oxa­penta­nediamide

**DOI:** 10.1107/S1600536809022806

**Published:** 2009-06-20

**Authors:** Shaohong Yin, Yu Cui, Guangpu Wu, Qi You, Guoxin Sun

**Affiliations:** a School of Chemistry and Chemical Engineering, University of Jinan, Jinan 250022, People’s Republic of China

## Abstract

In the title compound, C_18_H_20_N_2_O_3_, the two phenyl rings, adopt opposite orientations in the backbone and are oriented at a dihedral angle of 36.66 (3)°. In the crystal, inter­molecular C—H⋯O inter­actions link the mol­ecules into a three-dimensional network.

## Related literature

For a related structure, see: Zhang *et al.* (2001 ▶). For bond-length data, see: Allen *et al.* (1987 ▶).
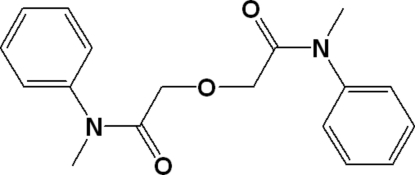


## Experimental

### 

#### Crystal data

C_18_H_20_N_2_O_3_
*M* *_r_* = 312.36
Monoclinic, 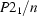

*a* = 10.7607 (11) Å
*b* = 10.7552 (12) Å
*c* = 14.7054 (14) Å
β = 102.897 (1)°
*V* = 1659.0 (3) Å^3^
*Z* = 4
Mo *K*α radiation
μ = 0.09 mm^−1^
*T* = 298 K
0.49 × 0.48 × 0.42 mm

#### Data collection

Bruker SMART CCD area-detector diffractometer
Absorption correction: multi-scan (*SADABS*; Sheldrick, 1996 ▶) *T*_min_ = 0.959, *T*_max_ = 0.965
8254 measured reflections
2897 independent reflections
1644 reflections with *I* > 2σ(*I*)
*R*_int_ = 0.082

#### Refinement

*R*[*F* ^2^ > 2σ(*F* ^2^)] = 0.049
*wR*(*F* ^2^) = 0.159
*S* = 1.04
2897 reflections
209 parameters
H-atom parameters constrained
Δρ_max_ = 0.18 e Å^−3^
Δρ_min_ = −0.17 e Å^−3^

### 

Data collection: *SMART* (Bruker, 2001 ▶); cell refinement: *SAINT* (Bruker, 2001 ▶); data reduction: *SAINT*; program(s) used to solve structure: *SHELXS97* (Sheldrick, 2008 ▶); program(s) used to refine structure: *SHELXS97* (Sheldrick, 2008 ▶); molecular graphics: *SHELXTL* (Sheldrick, 2008 ▶); software used to prepare material for publication: *SHELXTL*.

## Supplementary Material

Crystal structure: contains datablocks global, I. DOI: 10.1107/S1600536809022806/hk2708sup1.cif

Structure factors: contains datablocks I. DOI: 10.1107/S1600536809022806/hk2708Isup2.hkl

Additional supplementary materials:  crystallographic information; 3D view; checkCIF report

## Figures and Tables

**Table 1:** Hydrogen-bond geometry (Å, °)

*D*—H⋯*A*	*D*—H	H⋯*A*	*D*⋯*A*	*D*—H⋯*A*
C7—H7⋯O3^i^	0.93	2.53	3.436 (3)	165
C9—H9⋯O1^ii^	0.93	2.47	3.337 (3)	155
C12—H12*B*⋯O1^iii^	0.96	2.48	3.346 (3)	151
C17—H17⋯O2^iv^	0.93	2.52	3.432 (3)	167

Symmetry codes: (i) 
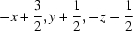
; (ii) 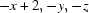; (iii) 
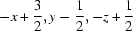
; (iv) 
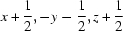
.

**Fig. 1.**
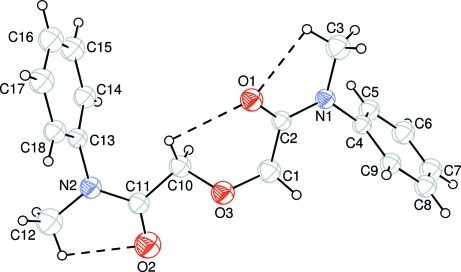
The molecular structure of the title molecule, with the atom-numbering scheme. Hydrogen bonds are shown as dashed lines.

## supplementary crystallographic information

### Comment

3-Oxapentanediamide derivatives show a highly selective complexation of lanthanide. We are interested in their performance to extract lanthanide ions. To obtain more information on the structural character and the reactivity of ligand with different lanthanide ions, we report herein the crystal structure of the title compound.

In the structure of the title compound (Fig 1), the bond lengths and angles  are within normal ranges (Allen *et al.*,  1987) and may be compared with the corresponding values in N,N'-diethyl-N,N'-diphenyl-3-oxapentanediamide (Zhang *et al.*,  2001). The framework of the molecule is composed of a zigzag chain (C2—C1—O3—C10—C11) with two methylphenyl amide terminal groups. An interesting aspect of the molecular conformation concerns the two phenyl rings, which adopt opposite orientations in the backbone, and they are oriented at a dihedral angle of 36.66 (3)°. The moieties (O1/O3/N1/C1-C3) and (O2/N2/C10-C13) are planar [with maximum deviations of -0.036 (3) and 0.021 (3) Å for atoms C3 and C10, respectively] and the dihedral angle between them is 24.67 (3)°, which are oriented with respect to the adjacent rings A (C4-C9) and B (C13-C18) at dihedral angles of 72.97 (4) and 70.17 (3) °, respectively. Intramolecular C-H···O interactions (Table 1) result in the formation of a six-membered ring C (O1/O3/C1/C2/C10/H10B) having twisted conformation, and two five-membered rings D (O1/N1/C2/C3/H3A) and E (O2/N2/C11/C12/H12C) having envelope conformations with atoms H3A and H12C displaced by -0.415 (4) and -0.257 (5) Å. In the crystal structure, intermolecular C-H···O interactions (Table 1) link the molecules into a three-dimensional network.

### Experimental

For the preparation of the title compound, a solution of diglycolic chloride (10 mmol) in anhydrous benzene (3 ml) was added dropwise to a mixture of *N*-methylphenylamine (25 mmol), anhydrous pyridine (2 ml) in anhydrous benzene (12.5 ml) in the ice-water bath. The mixture was stirred for 3 h, and then for another 3 h at room temperature. The crude product was recrystallized from toluene as the white solid (yield; 65%, m.p. 375 K). Crystals suitable for X-ray analysis were obtained from toluene by slow evaporation over a period of several days. C_18_H_20_N_2_O_3_: C 69.21, H 6.45, N 8.97%; found: C 69.09, H 6.32, N 8.78%. IR (KBr): *v* = 3056, 2973, 1666, 1593, 1497, 1120, 782, 704 cm ^-1^.

### Refinement

H atoms were positioned geometrically, with C-H = 0.93, 0.97 and 0.96 Å for aromatic, methylene and methyl H, respectively, and constrained to ride on their parent atoms, with U_iso_(H) = xU_eq_(C), where x = 1.5 for methyl H and x = 1.2 for all other H atoms.

### Crystal data

**Table:**

C_18_H_20_N_2_O_3_	*F*(000) = 664
*M**_r_* = 312.36	*D*_x_ = 1.251 Mg m^−^^3^
Monoclinic, *P*2_1_/*n*	Mo *K*α radiation, λ = 0.71073 Å
Hall symbol: -P 2yn	Cell parameters from 1991 reflections
*a* = 10.7607 (11) Å	θ = 2.7–21.6°
*b* = 10.7552 (12) Å	µ = 0.09 mm^−^^1^
*c* = 14.7054 (14) Å	*T* = 298 K
β = 102.897 (1)°	Block, yellow
*V* = 1659.0 (3) Å^3^	0.49 × 0.48 × 0.42 mm
*Z* = 4	

### Data collection

**Table:**

Bruker SMART CCD area-detector diffractometer	2897 independent reflections
Radiation source: fine-focus sealed tube	1644 reflections with *I* > 2σ(*I*)
graphite	*R*_int_ = 0.082
φ and ω scans	θ_max_ = 25.0°, θ_min_ = 2.1°
Absorption correction: multi-scan (*SADABS*; Sheldrick, 1996)	*h* = −12→12
*T*_min_ = 0.959, *T*_max_ = 0.965	*k* = −12→12
8254 measured reflections	*l* = −13→17

### Refinement

**Table:**

Refinement on *F*^2^	Secondary atom site location: difference Fourier map
Least-squares matrix: full	Hydrogen site location: inferred from neighbouring sites
*R*[*F*^2^ > 2σ(*F*^2^)] = 0.049	H-atom parameters constrained
*wR*(*F*^2^) = 0.159	*w* = 1/[σ^2^(*F*_o_^2^) + (0.0598*P*)^2^ + 0.3091*P*] where *P* = (*F*_o_^2^ + 2*F*_c_^2^)/3
*S* = 1.04	(Δ/σ)_max_ < 0.001
2897 reflections	Δρ_max_ = 0.18 e Å^−^^3^
209 parameters	Δρ_min_ = −0.17 e Å^−^^3^
0 restraints	Extinction correction: *SHELXS97* (Sheldrick, 2008), Fc^*^=kFc[1+0.001xFc^2^λ^3^/sin(2θ)]^-1/4^
Primary atom site location: structure-invariant direct methods	Extinction coefficient: 0.090 (9)

### Special details

**Table:**

Geometry. All e.s.d.'s (except the e.s.d. in the dihedral angle between two l.s. planes) are estimated using the full covariance matrix. The cell e.s.d.'s are taken into account individually in the estimation of e.s.d.'s in distances, angles and torsion angles; correlations between e.s.d.'s in cell parameters are only used when they are defined by crystal symmetry. An approximate (isotropic) treatment of cell e.s.d.'s is used for estimating e.s.d.'s involving l.s. planes.
Refinement. Refinement of *F*^2^ against ALL reflections. The weighted *R*-factor *wR* and goodness of fit *S* are based on *F*^2^, conventional *R*-factors *R* are based on *F*, with *F* set to zero for negative *F*^2^. The threshold expression of *F*^2^ > σ(*F*^2^) is used only for calculating *R*-factors(gt) *etc*. and is not relevant to the choice of reflections for refinement. *R*-factors based on *F*^2^ are statistically about twice as large as those based on *F*, and *R*- factors based on ALL data will be even larger.

### Fractional atomic coordinates and isotropic or equivalent isotropic displacement parameters (Å^2^)

**Table:**

	*x*	*y*	*z*	*U*_iso_*/*U*_eq_	
O1	0.95256 (18)	0.09798 (18)	0.10834 (12)	0.0572 (6)	
O2	0.5541 (2)	−0.1836 (2)	0.01992 (14)	0.0863 (8)	
O3	0.7658 (2)	−0.05186 (17)	0.01250 (12)	0.0628 (6)	
N1	0.94306 (19)	0.2313 (2)	−0.01069 (13)	0.0462 (6)	
N2	0.5968 (2)	−0.1632 (2)	0.17586 (15)	0.0557 (7)	
C1	0.7998 (3)	0.0548 (3)	−0.03176 (18)	0.0650 (9)	
H1A	0.7254	0.1077	−0.0500	0.078*	
H1B	0.8257	0.0297	−0.0881	0.078*	
C2	0.9057 (2)	0.1288 (2)	0.02810 (16)	0.0446 (7)	
C3	1.0382 (3)	0.3113 (3)	0.0461 (2)	0.0702 (9)	
H3A	1.0361	0.3012	0.1106	0.105*	
H3B	1.0202	0.3963	0.0281	0.105*	
H3C	1.1211	0.2892	0.0373	0.105*	
C4	0.9035 (2)	0.2600 (2)	−0.10801 (16)	0.0441 (7)	
C5	0.8270 (3)	0.3608 (3)	−0.1366 (2)	0.0569 (8)	
H5	0.7985	0.4094	−0.0931	0.068*	
C6	0.7922 (3)	0.3901 (3)	−0.2303 (2)	0.0705 (9)	
H6	0.7404	0.4587	−0.2496	0.085*	
C7	0.8333 (3)	0.3192 (3)	−0.2946 (2)	0.0664 (9)	
H7	0.8093	0.3393	−0.3576	0.080*	
C8	0.9092 (3)	0.2192 (3)	−0.26665 (19)	0.0607 (8)	
H8	0.9370	0.1708	−0.3107	0.073*	
C9	0.9458 (3)	0.1887 (3)	−0.17269 (18)	0.0527 (7)	
H9	0.9985	0.1207	−0.1536	0.063*	
C10	0.7034 (3)	−0.0267 (3)	0.08526 (18)	0.0586 (8)	
H10A	0.6570	0.0510	0.0734	0.070*	
H10B	0.7655	−0.0194	0.1440	0.070*	
C11	0.6129 (3)	−0.1313 (3)	0.09046 (19)	0.0546 (8)	
C12	0.5068 (3)	−0.2618 (3)	0.1840 (2)	0.0853 (11)	
H12A	0.4299	−0.2257	0.1951	0.128*	
H12B	0.5438	−0.3154	0.2350	0.128*	
H12C	0.4873	−0.3090	0.1272	0.128*	
C13	0.6656 (2)	−0.1065 (3)	0.26044 (17)	0.0462 (7)	
C14	0.6387 (3)	0.0131 (3)	0.2830 (2)	0.0600 (8)	
H14	0.5744	0.0576	0.2437	0.072*	
C15	0.7064 (3)	0.0669 (3)	0.3633 (2)	0.0710 (9)	
H15	0.6894	0.1485	0.3775	0.085*	
C16	0.7991 (3)	0.0009 (4)	0.4227 (2)	0.0725 (10)	
H16	0.8451	0.0376	0.4772	0.087*	
C17	0.8238 (3)	−0.1183 (3)	0.4018 (2)	0.0695 (9)	
H17	0.8863	−0.1634	0.4424	0.083*	
C18	0.7570 (3)	−0.1728 (3)	0.32081 (19)	0.0571 (8)	
H18	0.7739	−0.2546	0.3071	0.069*	

### Atomic displacement parameters (Å^2^)

**Table:**

	*U*^11^	*U*^22^	*U*^33^	*U*^12^	*U*^13^	*U*^23^
O1	0.0702 (13)	0.0581 (13)	0.0402 (11)	−0.0074 (10)	0.0056 (9)	0.0084 (9)
O2	0.1040 (18)	0.0934 (18)	0.0524 (13)	−0.0513 (15)	−0.0021 (12)	0.0040 (11)
O3	0.0947 (16)	0.0526 (12)	0.0479 (11)	−0.0247 (11)	0.0302 (10)	−0.0064 (9)
N1	0.0509 (13)	0.0468 (14)	0.0388 (12)	−0.0111 (11)	0.0058 (9)	0.0034 (10)
N2	0.0516 (14)	0.0659 (16)	0.0478 (14)	−0.0184 (12)	0.0072 (10)	0.0103 (11)
C1	0.083 (2)	0.067 (2)	0.0435 (16)	−0.0325 (18)	0.0119 (15)	0.0048 (14)
C2	0.0552 (16)	0.0431 (16)	0.0366 (14)	−0.0029 (14)	0.0128 (12)	0.0022 (12)
C3	0.079 (2)	0.072 (2)	0.0536 (18)	−0.0309 (18)	0.0008 (15)	0.0019 (15)
C4	0.0427 (14)	0.0477 (17)	0.0411 (14)	−0.0096 (13)	0.0076 (11)	0.0079 (12)
C5	0.0528 (17)	0.0564 (19)	0.0620 (18)	0.0019 (15)	0.0139 (13)	0.0108 (15)
C6	0.060 (2)	0.077 (2)	0.071 (2)	0.0115 (18)	0.0072 (16)	0.0285 (18)
C7	0.063 (2)	0.080 (2)	0.0493 (18)	−0.0150 (19)	−0.0014 (15)	0.0205 (17)
C8	0.072 (2)	0.066 (2)	0.0464 (16)	−0.0141 (18)	0.0179 (14)	0.0011 (15)
C9	0.0584 (17)	0.0523 (18)	0.0487 (16)	−0.0005 (14)	0.0145 (13)	0.0074 (13)
C10	0.077 (2)	0.0569 (19)	0.0455 (16)	−0.0217 (16)	0.0215 (14)	−0.0025 (13)
C11	0.0559 (17)	0.0565 (19)	0.0474 (16)	−0.0150 (15)	0.0026 (13)	0.0071 (14)
C12	0.079 (2)	0.103 (3)	0.072 (2)	−0.045 (2)	0.0125 (17)	0.0183 (19)
C13	0.0412 (15)	0.0555 (18)	0.0448 (15)	−0.0020 (13)	0.0157 (12)	0.0087 (13)
C14	0.0534 (18)	0.066 (2)	0.0649 (19)	0.0166 (16)	0.0217 (15)	0.0113 (16)
C15	0.087 (3)	0.066 (2)	0.069 (2)	0.0054 (19)	0.037 (2)	−0.0047 (18)
C16	0.084 (2)	0.086 (3)	0.0507 (18)	−0.010 (2)	0.0202 (17)	−0.0075 (18)
C17	0.069 (2)	0.085 (3)	0.0507 (18)	0.0095 (19)	0.0052 (15)	0.0092 (17)
C18	0.0613 (18)	0.0565 (19)	0.0524 (17)	0.0149 (15)	0.0100 (14)	0.0088 (14)

### Geometric parameters (Å, °)

**Table:**

O1—C2	1.221 (3)	C7—C8	1.358 (4)
O2—C11	1.225 (3)	C7—H7	0.9300
O3—C1	1.407 (3)	C8—C9	1.389 (4)
O3—C10	1.410 (3)	C8—H8	0.9300
N1—C2	1.343 (3)	C9—H9	0.9300
N1—C4	1.433 (3)	C10—C11	1.501 (4)
N1—C3	1.450 (3)	C10—H10A	0.9700
N2—C11	1.350 (3)	C10—H10B	0.9700
N2—C13	1.434 (3)	C12—H12A	0.9600
N2—C12	1.459 (4)	C12—H12B	0.9600
C1—C2	1.504 (4)	C12—H12C	0.9600
C1—H1A	0.9700	C13—C18	1.369 (4)
C1—H1B	0.9700	C13—C14	1.376 (4)
C3—H3A	0.9600	C14—C15	1.369 (4)
C3—H3B	0.9600	C14—H14	0.9300
C3—H3C	0.9600	C15—C16	1.368 (4)
C4—C5	1.370 (4)	C15—H15	0.9300
C4—C9	1.376 (4)	C16—C17	1.359 (5)
C5—C6	1.381 (4)	C16—H16	0.9300
C5—H5	0.9300	C17—C18	1.377 (4)
C6—C7	1.363 (4)	C17—H17	0.9300
C6—H6	0.9300	C18—H18	0.9300
			
C1—O3—C10	114.3 (2)	C4—C9—C8	119.5 (3)
C2—N1—C4	123.4 (2)	C4—C9—H9	120.2
C2—N1—C3	118.8 (2)	C8—C9—H9	120.2
C4—N1—C3	117.5 (2)	O3—C10—C11	108.6 (2)
C11—N2—C13	123.4 (2)	O3—C10—H10A	110.0
C11—N2—C12	119.1 (2)	C11—C10—H10A	110.0
C13—N2—C12	117.5 (2)	O3—C10—H10B	110.0
O3—C1—C2	113.7 (2)	C11—C10—H10B	110.0
O3—C1—H1A	108.8	H10A—C10—H10B	108.3
C2—C1—H1A	108.8	O2—C11—N2	121.5 (3)
O3—C1—H1B	108.8	O2—C11—C10	121.3 (2)
C2—C1—H1B	108.8	N2—C11—C10	117.2 (2)
H1A—C1—H1B	107.7	N2—C12—H12A	109.5
O1—C2—N1	122.4 (2)	N2—C12—H12B	109.5
O1—C2—C1	121.2 (2)	H12A—C12—H12B	109.5
N1—C2—C1	116.5 (2)	N2—C12—H12C	109.5
N1—C3—H3A	109.5	H12A—C12—H12C	109.5
N1—C3—H3B	109.5	H12B—C12—H12C	109.5
H3A—C3—H3B	109.5	C18—C13—C14	119.3 (3)
N1—C3—H3C	109.5	C18—C13—N2	119.9 (3)
H3A—C3—H3C	109.5	C14—C13—N2	120.8 (2)
H3B—C3—H3C	109.5	C15—C14—C13	120.2 (3)
C5—C4—C9	119.8 (2)	C15—C14—H14	119.9
C5—C4—N1	120.1 (2)	C13—C14—H14	119.9
C9—C4—N1	120.0 (2)	C16—C15—C14	120.2 (3)
C4—C5—C6	119.8 (3)	C16—C15—H15	119.9
C4—C5—H5	120.1	C14—C15—H15	119.9
C6—C5—H5	120.1	C17—C16—C15	119.8 (3)
C7—C6—C5	120.5 (3)	C17—C16—H16	120.1
C7—C6—H6	119.7	C15—C16—H16	120.1
C5—C6—H6	119.7	C16—C17—C18	120.4 (3)
C8—C7—C6	119.9 (3)	C16—C17—H17	119.8
C8—C7—H7	120.0	C18—C17—H17	119.8
C6—C7—H7	120.0	C13—C18—C17	120.0 (3)
C7—C8—C9	120.4 (3)	C13—C18—H18	120.0
C7—C8—H8	119.8	C17—C18—H18	120.0
C9—C8—H8	119.8		
			
C10—O3—C1—C2	70.1 (3)	C1—O3—C10—C11	149.2 (2)
C4—N1—C2—O1	170.6 (2)	C13—N2—C11—O2	178.6 (3)
C3—N1—C2—O1	−2.9 (4)	C12—N2—C11—O2	−0.4 (4)
C4—N1—C2—C1	−11.0 (4)	C13—N2—C11—C10	−2.9 (4)
C3—N1—C2—C1	175.4 (3)	C12—N2—C11—C10	178.1 (3)
O3—C1—C2—O1	−2.1 (4)	O3—C10—C11—O2	−36.2 (4)
O3—C1—C2—N1	179.5 (2)	O3—C10—C11—N2	145.2 (3)
C2—N1—C4—C5	113.5 (3)	C11—N2—C13—C18	−109.3 (3)
C3—N1—C4—C5	−72.9 (3)	C12—N2—C13—C18	69.7 (3)
C2—N1—C4—C9	−68.7 (3)	C11—N2—C13—C14	72.3 (3)
C3—N1—C4—C9	104.9 (3)	C12—N2—C13—C14	−108.7 (3)
C9—C4—C5—C6	0.3 (4)	C18—C13—C14—C15	2.6 (4)
N1—C4—C5—C6	178.1 (2)	N2—C13—C14—C15	−179.0 (2)
C4—C5—C6—C7	0.1 (4)	C13—C14—C15—C16	−1.6 (4)
C5—C6—C7—C8	−0.2 (5)	C14—C15—C16—C17	−0.1 (5)
C6—C7—C8—C9	−0.2 (4)	C15—C16—C17—C18	0.6 (5)
C5—C4—C9—C8	−0.7 (4)	C14—C13—C18—C17	−2.1 (4)
N1—C4—C9—C8	−178.5 (2)	N2—C13—C18—C17	179.5 (2)
C7—C8—C9—C4	0.6 (4)	C16—C17—C18—C13	0.4 (4)

### Hydrogen-bond geometry (Å, °)

**Table:**

*D*—H···*A*	*D*—H	H···*A*	*D*···*A*	*D*—H···*A*
C3—H3A···O1	0.96	2.36	2.707 (3)	101
C7—H7···O3^i^	0.93	2.53	3.436 (3)	165
C9—H9···O1^ii^	0.93	2.47	3.337 (3)	155
C10—H10B···O1	0.97	2.53	2.949 (3)	106
C12—H12B···O1^iii^	0.96	2.48	3.346 (3)	151
C12—H12C···O2	0.96	2.31	2.709 (3)	104
C17—H17···O2^iv^	0.93	2.52	3.432 (3)	167

Symmetry codes: (i) −*x*+3/2, *y*+1/2, −*z*−1/2; (ii) −*x*+2, −*y*, −*z*; (iii) −*x*+3/2, *y*−1/2, −*z*+1/2; (iv) *x*+1/2, −*y*−1/2, *z*+1/2.
